# Implications of Acute Kidney Injury Requiring Dialysis Versus End-Stage Kidney Disease Status

**DOI:** 10.1053/j.ajkd.2025.11.015

**Published:** 2026-03-23

**Authors:** Ian E. McCoy, Eugene Lin

**Affiliations:** Division of Nephrology, University of California, San Francisco, San Francisco (IEM), Division of Nephrology, Departments of Medicine and Health Policy & Management, Keck School of Medicine of USC and USC Sol Price School of Public Policy (EL), and Leonard D. Schaeffer Center for Health Policy & Economics, University of Southern California, Los Angeles (EL), California.

## Abstract

More than 20% of patients receiving dialysis at in-center hemodialysis facilities initiate dialysis for acute kidney injury requiring dialysis (AKI-D) rather than end-stage kidney disease (ESKD). The transition from AKI-D to ESKD status is important to reorient clinical efforts from short-term monitoring for recovery to equally important long-term goals of establishing permanent dialysis access and planning for kidney transplant. The timing of this transition is highly variable and determined by the treating nephrologist. Because of numerous subtle policy differences, the implications of transitioning a patient receiving dialysis for AKI-D to ESKD status can be complex and far-reaching. Here, we aim to describe the financial and nonfinancial implications of the AKI-D–to–ESKD status transition. Clinicians and policymakers alike may be able to improve the care of this vulnerable patient population through a better understanding of these implications. We conclude with policy recommendations to address disincentives to optimal care.

## Introduction

**Figure 1.**
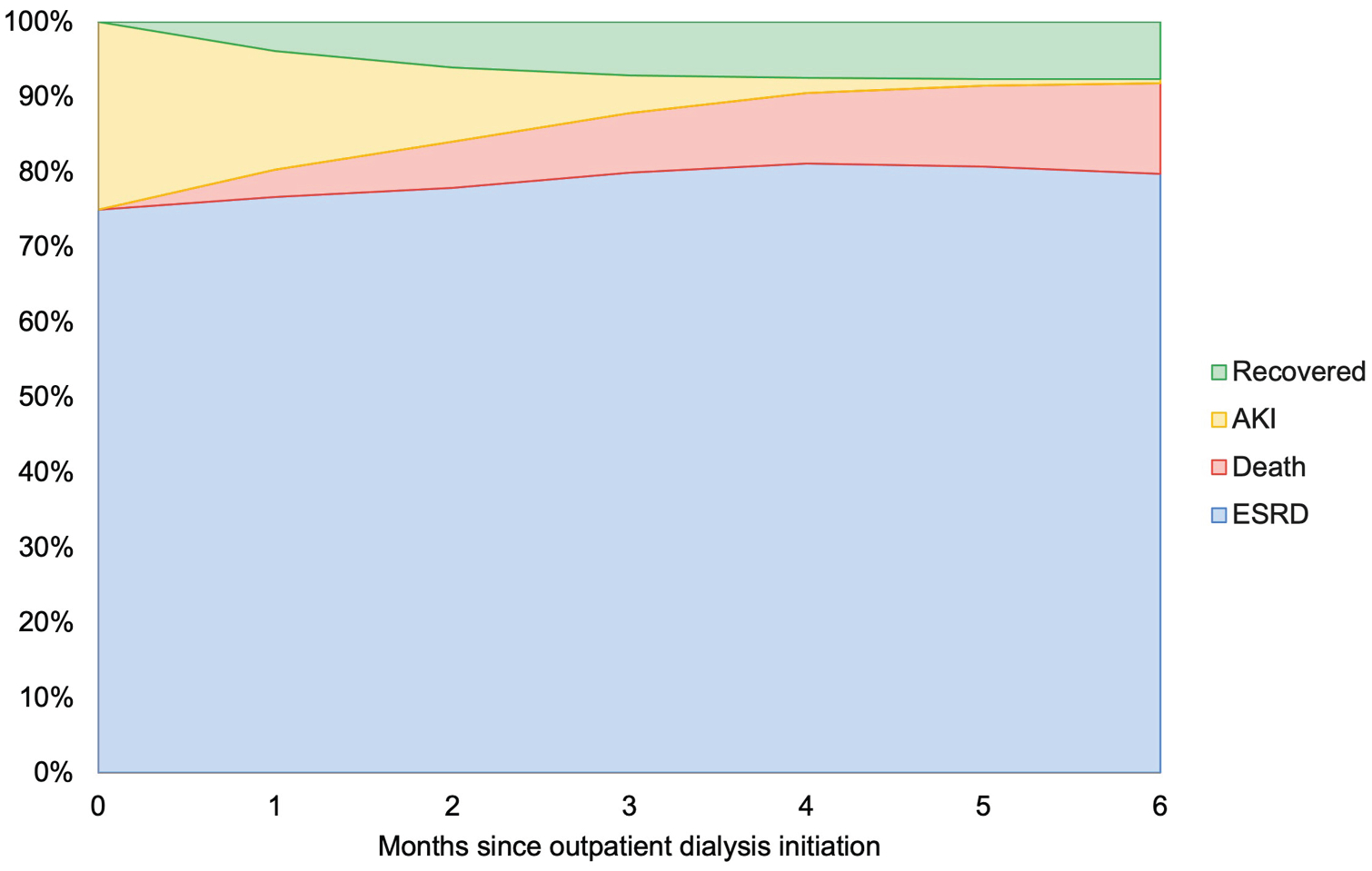
Changes in classification of an incident hemodialysis population during an interval of 6 months, including end-stage kidney disease (ESKD) and acute kidney injury requiring dialysis (AKI-D). Simulated data combining reported outcomes in the AKI-D and ESKD populations (assumes 3 patients with incident ESKD for every one with incident AKI-D, linear cumulative ESKD mortality rate of 10% at 6 months, and AKI-D outcomes per figure 4.10 of the 2024 US Renal Data System Annual Data Report^8^).

Acute kidney injury requiring dialysis (AKI-D) constitutes >20% of outpatient hemodialysis initiations.^1–5^ When patients with AKI-D are deemed unlikely to recover enough kidney function to discontinue dialysis, they are transitioned from AKI-D to end-stage kidney disease (ESKD) status. The timing of this transition is usually left to the treating nephrologist,^6,7^ who is best positioned to predict the likelihood of recovery based on the level of pre-AKI kidney function, the cause of AKI, comorbid conditions, and other factors. In practice, the transition from AKI-D to ESKD status is highly variable, occurring any time from a few weeks after the injury to more than 6 months after, although most patients experience the transition by 90 days (Fig 1). Ideally, this variation stems entirely from clinical differences in patients’ likelihoods of recovery (ie, transitioning to ESKD more quickly for patients less likely to recover) in a patient-centered approach.

However, because the transition from AKI-D to ESKD status is subjective, it leaves open the possibility that some of this heterogeneity in timing may be influenced by factors that are orthogonal to the patient’s clinical trajectory such as idiosyncratic practice patterns. It is important to ensure that policy and unintended financial incentives have minimal influence on this clinical decision making, both to preserve good care as well as to avoid any perception of potential conflicts of interest that could erode trust between patients and providers. Patients should be confident that their care team will take a patient-centered approach that ensures their residual kidney function and clinical trajectory are carefully followed. Patients must also trust that their providers are closely monitoring for kidney recovery so, when they do transition to ESKD status, they can be reoriented from short-term monitoring for recovery to the equally important long-term goals of establishing permanent dialysis access and planning for kidney transplant.

Nephrologists may not be aware that the timing of the transition to ESKD status may have important side effects on patient care because of numerous subtle policy differences. This piece aims to describe the financial and nonfinancial implications of the AKI-D–to–ESKD status transition and present policy recommendations to improve the perverse incentives that currently exist.

## When Does the AKI-D–to–ESKD Transition Occur?

Data on outpatient AKI-D management, including the timing of the transition to ESKD status, are limited. Although the US Renal Data System reports on all patients with Medicare Fee-For-Service (ie, traditional Medicare) in the 100% Medicare AKI-D database,^8^ it does not capture patients with Medicare Advantage, Medicaid, private insurance, or those without insurance. The US Renal Data System also does not include the 100% AKI database on its list of available Standard Analysis Files for researchers, who are limited to the 5% chronic kidney disease sample except for patients with AKI-D in whom ESKD eventually develops. The data we do have indicate substantial heterogeneity in the timing of the transition to ESKD status. Some patients go through the transition within a few weeks, whereas others maintain AKI-D status beyond 6 months of dialysis. The US Renal Data System reports that, among traditional Medicare beneficiaries who initiated outpatient dialysis for AKI-D in 2020 and continued to receive dialysis at 3 months, 66% had transitioned to ESKD status, increasing to 97% at 6 months. Using 2018 data, Fresenius Medical Care, which supplies more than one third of outpatient dialysis treatments in the United States, showed that, among patients who continued to receive dialysis at 90 days, 79% who initiated dialysis for AKI-D had transitioned to ESKD status.^9^ That figure increased to >98% by 150 days. It is unknown to what extent the timing of the transition relates to clinical and/or biological factors (eg, residual renal function) versus other considerations.

## AKI-D Does Not Automatically Confer Medicare Eligibility

Because ESKD confers Medicare eligibility (after a coordination-of-benefits period),^10^ transitioning patients from AKI-D to ESKD status has important financial implications for dialysis facilities, especially if it results in patients switching payers (Table 1). On average, dialysis facilities break even^11^ or run a negative profit margin for Medicare-financed dialysis. DaVita^12^ reports that virtually all its profits come from the minority of patients who have private insurance^13,14^ in the form of employer group health plans or Affordable Care Act individual market plans. Assuming that dialysis facilities have no margin on traditional Medicare, the average per-treatment cost of thrice-weekly in-center dialysis is approximately equal to the Medicare payment.^15^ A crude back-of-the-envelope calculation using the base rate of $273.83 per treatment puts the annualized average cost of dialysis at approximately $37,950 after accounting for missed treatments (approximately 1.45 treatments per month^16^). Because the Affordable Care Act and other private plans pay as much as 3–6 times more for dialysis,^16,17^ facilities can therefore earn a profit margin of $75,900-$189,750 per patient per year. Thus, transitioning patients with private insurance plans from AKI-D to ESKD status could result in facilities foregoing large profit margins if it accelerates the switch from private insurance to Medicare. Conversely, because Medicaid pays substantially less than Medicare, providers have financial incentives to transition patients with Medicaid to ESKD status early. In ESKD, 8% of incident hemodialysis recipients have an employer group health plan^18^; the corresponding percentage in AKI-D is unknown. Patients’ out-of-pocket dialysis costs also differ among insurance plans (patients are responsible for 20% of dialysis costs under Medicare Part B, which may be defrayed by Medicaid or other supplemental insurance), which could be a factor in whether early transition to Medicare is beneficial for a particular patient.^19,20^

## Facilities Do Not Receive the Incident Patient Multiplier for Patients With AKI-D

In recognition of the higher costs of dialysis initiation compared with dialysis continuation, facilities receive a higher payment during the first 120 days of dialysis for patients with ESKD, known as the incident ESKD patient multiplier. This multiplier is the largest positive adjustment to the base rate in the prospective payment system.^21^ Patients with AKI-D, however, do not receive the multiplier until they eventually transition to ESKD status. Revenue from the multiplier is lost among patients with AKI-D who never transition to ESKD status (as a result of death or recovery of kidney function). Thus, facilities may favor earlier transition to ESKD status to capture the multiplier.

## Options for More Than 3 Treatments per Week

In response to feedback from dialysis stakeholders,^22^ the US Centers for Medicare and Medicaid Services allow facilities to bill more than 3 treatments per week without medical justification, unlike in ESKD.^15^ Therefore, there may be a financial advantage for dialysis facilities to delay the transition to ESKD status in patients who require more than 3 treatments per week, especially if the facility has empty chairs in their “off shifts.” Similarly, nephrologists managing AKI-D may also bill more than 4 times per month, but doing so would disrupt nephrologists’ rounding schedules, which revolve around ESKD. It is not entirely clear if this flexibility materializes significant financial benefit to facilities: currently, only 1% of patients with AKI-D receive treatment at a frequency greater than 3 times per week.^5^ Most patients with AKI-D who are not on a thrice-weekly schedule are receiving less frequent dialysis, not more.

## AKI-D–Specific Costs Are Not Separately Reimbursed

Patients with AKI-D incur the additional costs of recovery monitoring (timed urine collections and additional blood-work) and customized treatments with respect to dialysis prescriptions, ultrafiltration rates, and medication dosages.^23^ Ideally, Medicare’s AKI-D payment system allows for this clinically appropriate customization of dialysis care.^15^ However, facilities are disincentivized from monitoring residual renal function closely because the Centers for Medicare and Medicaid Services pay for all dialysis-related services in a per-treatment bundle irrespective of whether the patient has AKI-D or ESKD (ie, there is no extra payment to compensate for the costs of recovery monitoring). Additionally, there are system costs to customizing protocols for the minority of patients with AKI-D. Research in other sectors of health care demonstrate that multitasking and task-switching may result in errors and worse outcomes.^24^ AKI-D represents only a small fraction of most facilities’ patients (>20% of incident cases but <5% of prevalent cases).^25^ Therefore, it may be more profitable for facilities to ignore issues specific to AKI-D because it is operationally simpler to manage all patients as if they had ESKD. Unfortunately, recent research has demonstrated that facilities essentially treat patients with AKI-D identically to those with ESKD.^5^ Because nurses and dialysis technicians must care for multiple patients at once, it may be too costly for facilities to customize dialysis treatments for AKI-D, at least with the current payment structure.

## Patients With AKI-D Are Excluded From Quality Metrics

Additionally, the Centers for Medicare and Medicaid Services’ quality measures^26–28^ only hold facilities accountable for patients with ESKD.^29^ Facilities may avoid financial penalties by keeping patients designated as having AKI-D. For instance, patients who are poor candidates for an arteriovenous fistula or graft or who are receiving lower intensities of dialysis (ie, single-pool Kt/V <1.2) could be kept with AKI-D status to avoid penalties. Finally, facilities enrolled in the Kidney Care Choices model^30,31^ are penalized if patients do not experience an “optimal start,” defined as starting dialysis as an outpatient with an arteriovenous fistula or graft. Even if providers do not actively game these metrics, the lack of accountability leaves the impression that it could be profitable to do so.

## AKI-D Status Results in Variable Physician Reimbursement

Nephrologists are free to bill different service codes depending on their idiosyncratic billing practices and where they see patients (ie, in the dialysis facility vs in the clinic). If the nephrologist uses standard evaluation and management clinic codes, nephrologists could be reimbursed at a higher level for AKI-D than for ESKD on a monthly basis, assuming they bill more than 4 times per month. However, many nephrologists likely use Current Procedural Terminology code 90935, which pays at a much lower rate than the ESKD monthly capitated payment (Current Procedural Terminology codes 90960–90962, which do not apply to AKI-D) or a level 5 office visit, even if the nephrologist sees the patient 4 times per month.^21^

Although it is theoretically possible for nephrologists to assess patients more than 4 times per month and get paid for doing so, this practice is unlikely to be financially worthwhile as a result of the opportunity cost on time. Seeing patients more often would require the managing nephrologist to make dedicated trips to the dialysis facility specifically for patients with AKI-D. Such a practice would be extremely time-consuming. Because patients with AKI-D constitute a small minority of a nephrologist’s patient volume, the additional revenue is unlikely to outweigh the loss of revenue from foregoing other revenue-generating practices.

## Nonfinancial Implications of AKI-D Versus ESKD Status

Although the distinct clinical needs of patients with AKI-D have been described in detail elsewhere,^23,32,33^ here we will highlight a few of the most important clinical implications of the transition to ESKD status (Table 2). First, the transition to ESKD status has some nonfinancial policy implications for patient care. For example, patients do not begin accruing time on the deceased-donor transplant waitlist until the date of ESKD (not the date of first dialysis/AKI-D).^34^ In addition, until a recent change in policy that became active in 2025, patients with AKI-D were also not eligible for home dialysis, a therapy that may be particularly helpful for the subset of motivated patients with AKI-D who can closely monitor their urine output recovery and adjust their dialysis intensity to avoid recurrent AKI via intradialytic hypotension.

Outside of formal policy, AKI-D status also focuses clinical resources on different goals. AKI-D status signals to the clinical team that monitoring for renal recovery is the highest priority, with recommended weekly blood work and timed urine collections.^23^ Residual kidney function can change rapidly in AKI-D, which may require frequent changes in the intensity of the dialysis prescription or medications. Delayed recognition of improved residual kidney function leads to unnecessary dialysis and could inadvertently result in recurrent AKI via dialytrauma^35^ and preventable cases of ESKD. By contrast, the transition to ESKD status tells the clinical team to shift focus to long-term goals including transitioning from a dialysis catheter to a more permanent arteriovenous fistula with less risk of infection and being referred to a transplant center for possible living-donor kidney transplant. The timing of the transition to ESKD status will never be perfect, and some patients may recover from AKI-D beyond 6 months,^36^ so monitoring for recovery should continue after the transition to ESKD status, albeit at reduced intensity.

## Implications and Policy Recommendations

Nephrologists may or may not be aware of all of these implications. Although there are virtually no research data available regarding nephrologists’ practice patterns in determining the AKI-D–to–ESKD transition, our intuition is that most nephrologists decide on AKI-D versus ESKD status based on clinical factors alone, rather than factors extrinsic to clinical care. Still, it is important for clinicians to understand how policy surrounding the AKI-D/ESKD status decision may materially impact patients.

As we have reviewed above, current policy has not yielded customized or ideal AKI-D care. Despite recognition by expert panels^23^ and the federal government^37^ that AKI-D treatment should be substantially different from ESKD treatment, most patients with AKI-D receive treatment that is virtually indistinguishable from that received by patients with ESKD, including thrice-weekly treatment frequency for >93%.^5^ Only a minority of patients complete the recommended timed urine collections to monitor for recovery.^5^

Policymakers should address the following problems (Table 3): (1) no incentives to monitor patients for recovery, (2) no incentives to treat AKI-D differently than ESKD, (3) no accountability for AKI-D outcomes because they are excluded from quality metrics, and (4) no transplant wait-time accrual until ESKD.

First, the most important issue with current AKI-D policy, in our opinion, is that facilities and nephrologists have no financial incentive to search for and correctly identify recovery. Currently, the time and effort required to test for recovery is not compensated, and successfully weaning and safely discontinuing dialysis results in a complete loss of revenue for dialysis providers and decreased revenue for nephrologists. Additionally, clinicians lose a multidisciplinary team of nurses, dieticians, and social workers when they deem a patient to have recovered sufficient kidney function to discontinue dialysis. These financial hurdles may limit the ability of conscientious providers to carve out the extra time required to detect subtle, partial recoveries (ie, recovery to a level just above that at which dialysis can be safely weaned or discontinued) and push against the inertia of continuing dialysis indefinitely. In contemporary practice, recommended timed urine collections are completed in fewer than one third of patients,^5^ and kidney recovery is missed at an unknown rate.^38–40^ Every missed opportunity to discontinue dialysis in a patient who has recovered adequate kidney function results in tens of thousands of wasted health care dollars per patient and, more importantly, worsened patient quality of life and outcomes. Even if the financial disincentive does not affect provider behavior, at minimum, its mere presence may erode trust by creating the perception that profitability is at odds with high-quality care.

One way to incentivize appropriate dialysis discontinuation is to financially reward dialysis providers and nephrologists for each case in which dialysis is discontinued as a result of renal recovery. A straightforward way to implement this could be through a quality measure or value-based care model. Although such a financial bonus could create a new perverse incentive to discharge inpatients with AKI-D to outpatient dialysis earlier, before recovery occurs, to capture the outpatient recovery bonus, this concern could be mitigated by limiting the recovery bonus to cases in which recovery was not trivial to uncover and manage (eg, dialysis discontinuation when the timed urine creatinine clearance is <20 mL/min).

Second, AKI-D care is not paid at parity with ESKD care because of unreimbursed costs of recovery monitoring and customized care. AKI-D is also excluded from the incident ESKD patient multiplier. To ensure that facilities are not punished for performing recommended monitoring, we suggest unbundling the costs of additional laboratory testing specific to AKI-D (ie, reimburse the costs of recommended weekly timed urine collections and basic metabolic panels).^23^ Although this would be an important intermediate step in correcting perverse incentives in AKI-D care, this change alone would not bridge the financial shortfalls of facilities that choose to invest in developing the robust AKI-D treatment infrastructure necessary to bring recovery monitoring efforts up to recommended levels. There is a significant operational cost to treating any subgroup of patients (in this case, those with AKI-D) differently. Such costs may exceed the small financial incentives enumerated in Table 1 and may be the biggest reason that patients with AKI-D currently receive the same treatment as those with ESKD. Therefore, we also recommend that the incident ESKD patient multiplier be extended to AKI-D. To avoid the perverse incentive to keep patients designated as having AKI-D for 120 days before transitioning to ESKD status, we suggest that this multiplier apply to the first 120 days of dialysis, irrespective of whether dialysis is for AKI-D or ESKD.

Third, we recognize the reasons that patients with AKI-D have been excluded from quality metrics. Namely, AKI-D quality metrics are challenging to implement because of the lack of evidence-based guidelines for outpatient AKI-D care, the heterogeneity of AKI-D, and the small patient volume of AKI-D at the average dialysis unit. Nevertheless, recovery monitoring is universally recommended as important in AKI-D care and could be easily tracked via laboratory orders. Therefore, we suggest that the Centers for Medicare and Medicaid Services explore the construction of an AKI-D–specific quality metric examining the percentage of patients with AKI-D who complete a timed urine collection during the first 30 days of outpatient dialysis. In addition to improving patient care at an individual level, ascertaining such data on the level of residual kidney function at which recovery and ESKD are currently declared may facilitate future guideline development.

Fourth, patients who remain with AKI-D status are penalized by not accruing time on the transplant waitlist until they are transitioned to ESKD status (the loss of wait time averages 3 months). Because the timing of the AKI-D–to–ESKD status transition is somewhat arbitrary and the requirement for dialysis already implies an estimated glomerular filtration rate <20 mL/min/1.73 m^2^, we suggest that a more equitable policy would use the overall start date of dialysis rather than the start date of ESKD to determine wait time accrued for a transplant. This change would remove this penalty without causing unnecessary listings for patients with AKI-D whose kidney function recovers.

## Conclusions

In conclusion, the downstream consequences of transitioning a patient receiving dialysis for AKI-D to ESKD status are complex and far-reaching. Clinicians and policy makers alike may be able to improve the care of this vulnerable patient population through a better understanding of these implications.

## Figures and Tables

**Table 1.** Financial Implications of AKI-D Versus ESKD Status Designation

AKI Payment Regulation	Purpose	Operational Challenges	Likely Net Financial Incentive for AKI-D
AKI-D status does not result in Medicare coverage (until transitioning to ESKD)	Medicare coverage is only an entitlement for ESKD		Depends on baseline insurance status, but likely +++ facility and + nephrologist if privately insured
HD for AKI-D can be reimbursed more than 3 times per week	Allows for customized dialysis	Offering a fourth and fifth treatment is costly if it takes up a chair	+/− Facility: potential increase revenue for the extra treatments, but not if it disrupts scheduling of other thrice weekly patients; (no effect) nephrologist
Nephrologists may bill for as many visits as needed	Allows for closer monitoring	Nephrologists must make an extra trip to the dialysis facility	(No effect) Facility; +/− nephrologist (depends on opportunity cost of time)
Patients with AKI-D are exempt from quality measurement	Allows for facilities to focus on renal recovery	May reduce accountability for AKI-D outcomes	+ Facility; (no effect) nephrologist
Base payment for AKI-D is not adjusted for incident ESKD patient multiplier	Incident multiplier recognizes that incident cases are costlier to treat than prevalent cases		− Facility: lost for patients who recover or die before ESKD, delayed for those who progress to ESKD; (no effect) nephrologist
Monthly capitated ESKD CPT codes (90960–90962) do not apply to AKI-D	ESKD codes limited to 4 visits per month	Many nephrologists may bill using 90935 CPT code, which is reimbursed at a lower level than the 90962 code, but others may be billing higher-reimbursement clinic visits such as 99215	(No effect) Facility; +/− nephrologist depending on code used and frequency of visits

Abbreviations: AKI-D, acute kidney injury requiring dialysis; CPT, Current Procedural Terminology; ESKD, end-stage kidney disease.

**Table 2.** Nonfinancial Implications of AKI-D Versus ESKD Status Designation

Distinction	Purpose	Operational Challenges	Likely Net Incentive
AKI-D status does not accrue time on the transplant waitlist	A large minority of AKI-D patients recover, making transplant listing unnecessary	Currently, AKI-D start date (and most AKI-D characteristics) is not uniformly collected like ESKD start date by USRDS	May encourage nephrologists to convert to ESKD status earlier
AKI-D patients need closer monitoring, including weekly blood work and timed urine collection	The residual kidney function may change rapidly in AKI-D	No additional reimbursement to offset the increased costs of ordering, processing, and interpreting these tests, not to mention increased infrastructure to develop robust, distinct treatment pathways for AKI-D vs ESKD in the dialysis unit	May encourage earlier transition to ESKD to minimize additional costs and testing burden
AKI-D patients need dynamic volume status assessment and UF goals rather than more stable dry weights	AKI-D patients may have rapidly changing urine output, which puts them at risk for recurrent AKI from intradialytic hypotension due to excessive UF	Unclear treatment goal for dialysis staff, who typically view higher achieved UF as a success to help keep their patients euvolemic; special training required to recognize risks of excessive UF in AKI-D	May encourage earlier transition to ESKD to simplify dialysis prescription (eg, to minimize calls to clarify moving UF targets)
ESKD status may be viewed by patients as “giving up hope”	ESKD status helps communicate to the patient and treatment team that the clinical focus has shifted to long-term treatment goals like dialysis access, adequacy, and transplant workup; this does not mean that nephrologists completely stop evaluating for recovering kidney function	Nephrologists may not want to “be the bad guy” to tell a patient that they are most likely not going to recover and it now makes sense to shift the focus of clinical resources to long-term dialysis/transplant/hospice planning	May encourage prolonged AKI-D status
AKI-D patients may be reluctant to have permanent access (AVF/AVG) placed while kidney recovery is still believed likely	Almost all AKI-D patients undergo hemodialysis via tunneled dialysis catheters	Dialysis catheters have a higher risk of bloodstream infection vs AVFs/AVGs	May encourage earlier transition to ESKD to protect patients from catheter infections

Abbreviations: AKI-D, acute kidney injury requiring dialysis; AVF, arteriovenous fistula; AVG, arteriovenous graft; CPT, Current Procedural Terminology; ESKD, end-stage kidney disease; UF, ultrafiltration; USRDS, US Renal Data System.

**Table 3.** Policy Recommendations for Outpatient AKI-D

Recommendation	Purpose	Potential Unintended Consequences	Ways to Mitigate
Provide a recovery bonus for each case in which dialysis is discontinued because of renal recovery	Recognizes time/effort of recovery monitoring and careful dialysis weaning/discontinuation as well as associated loss of revenue	Could incentivize earlier hospital discharge so outpatient recovery bonus can be captured	Limit bonus to cases in which recovery was not trivial to uncover and manage (eg, dialysis discontinuation when timed urine creatinine clearance is <20 mL/min)
Unbundle costs of additional laboratory testing specific to AKI-D (ie, reimburse costs of weekly timed urine collections and basic metabolic panels)	Avoids punishing facilities that perform recommended monitoring tests	Could incentivize unnecessary laboratory testing in patients who are maintained as having AKI-D despite low likelihood of recovery as determined by nephrologist	Limit to 90 d
Extend the incident ESKD patient multiplier to AKI-D	Recognizes increased costs of treating a subgroup of patients with a distinct care pathway (eg, AKI-D or incident ESKD)	Could incentivize keeping patients designated with AKI-D for 120 d before transitioning to ESKD	Multiplier should apply to the first 120 d of dialysis irrespective of whether dialysis is for AKI-D or ESKD
Create a quality metric assessing the percentage of AKI-D patients who complete a timed urine collection during the first 30 d of outpatient dialysis	Begins to track quality care in recommended recovery monitoring	May be statistically noisy if facilities do not have many patients with AKI-D	Assess only facilities with at least a minimum number of patients; assess performance at the chain/ownership level rather than facility level
Use the overall start date of dialysis rather than the start date of ESKD to determine wait time accrued for transplant	Stops penalizing patients who may be appropriately kept with AKI-D status	None apparent	NA

Abbreviations: AKI-D, acute kidney injury requiring dialysis; ESKD, end-stage kidney disease; NA, not applicable.

## References

[1] McCoy I, Hsu CY. Dialyzing acute kidney injury patients after hospital discharge. Clin J Am Soc Nephrol. 2021;16(6):848–849. doi:10.2215/CJN.04590421 34117078 PMC8216627

[2] Dahlerus C, Segal J, He K, Acute kidney injury requiring dialysis and incident dialysis patient outcomes in U.S. outpatient dialysis facilities. Clin J Am Soc Nephrol. 2021;16(6):853–861. 34045300 10.2215/CJN.18311120 PMC8216606

[3] Dalrymple L New insights on acute kidney injury. Fresenius Medical Care North America Medical Office Live; 2019. Accessed October 2, 2022. https://www.youtube.com/watch?v=faszltBUvbQ

[4] Caring for AKI patients at DaVita^®^; 2019. Accessed August 2, 2023. https://www.davita.com/-/media/davita/project/kidneycare/pdf/partners/caring-for-aki-patients-at-davita.ashx

[5] McCoy IE, Weinhandl E, Hussein W, Hsu CY. Initial management and potential opportunities to deprescribe dialysis among patients with AKI-D patients after hospital discharge. J Am Soc Nephrol. 2023;34(12):1949–1951. doi:10.1681/asn.0000000000000225 37768189 PMC10703077

[6] Heung M, Faubel S, Watnick S, Outpatient dialysis for patients with AKI. Clin J Am Soc Nephrol. 2015;10(10):1868–1874. doi:10.2215/CJN.02290215 26220818 PMC4594066

[7] Heung M Outpatient Dialysis for acute kidney injury: progress and pitfalls. Am J Kidney Dis. 2019;74(4):523–528. doi:10.1053/j.ajkd.2019.03.431 31204193

[8] Johansen KL, Gilbertson DT, Li S, US Renal Data System 2024 Annual Data Report: epidemiology of kidney disease in the United States. Am J Kidney Dis. 2025;85(6)(suppl 1):A8–A11. doi:10.1053/j.ajkd.2025.02.602 40379355

[9] Dalrymple LS, Ofsthun NJ. Acute kidney injury necessitating outpatient dialysis. Fresenius Medical Care North America. Accessed February 11, 2025. https://freseniusmedicalcare.com/en-us/insights/amr/2019/acute-kidney-injury-necessitating-outpatient-dialysis

[10] Centers for Medicare and Medicaid Services. End-stage renal disease (ESRD). Accessed April 13, 2025. https://www.medicare.gov/basics/end-stage-renal-disease

[11] Medicare Payment Advisory Commission. Chapter 5: outpatient dialysis services, figure 5–5. Report to the Congress: Medicare Payment Policy; March 2025. Accessed April 13, 2025. https://www.medpac.gov/wp-content/uploads/2025/03/Mar25_Ch5_MedPAC_Report_To_Congress_SEC.pdf

[12] DaVita Annual Report; 2023. Accessed April 13, 2025. https://investors.davita.com/download/DaVita_2023_Annual_Report.pdf

[13] Lin E The cost of transferring dialysis care from the employer-based market to Medicare. JAMA Netw Open. 2021;4(3):e212113. doi:10.1001/jamanetworkopen.2021.2113 33734412 PMC7974636

[14] Lin E, Erickson KF. Payer mix among patients receiving dialysis. JAMA. 2020;324(9):900. doi:10.1001/jama.2020.10771 32870292

[15] Centers for Medicare & Medicaid Services. Final rule; November 12, 2024. Accessed April 13, 2025. https://public-inspection.federalregister.gov/2024-25486.pdf

[16] Lin E, McCoy MS, Liu M, Association between nephrologist ownership of dialysis facilities and clinical outcomes. JAMA Intern Med. 2022;182(12):1267. doi:10.1001/jamainternmed.2022.5002 36342723 PMC9641593

[17] League RJ, Eliason P, McDevitt RC, Roberts JW, Wong H. Variability in prices paid for hemodialysis by employer-sponsored insurance in the US from 2012 to 2019. JAMA Netw Open. 2022;5(2):e220562. doi:10.1001/jama-networkopen.2022.0562 35226088 PMC8886517

[18] United States Renal Data System. ESRD reference table D; 2024. Accessed April 14, 2025. https://usrds-adr-api.niddk.nih.gov/api/referenceTables?year=2024&referenceTable=ESRD_Ref_D_Modality_2024

[19] American Kidney Fund. Insurance and costs for dialysis. Accessed May 22, 2025. https://www.kidneyfund.org/resource/insurance-and-costs-dialysis

[20] League RJ, Eliason P, McDevitt RC, Roberts JW, Wong H. Assessment of spending for patients initiating dialysis care. JAMA Netw Open. 2022;5(10):e2239131. doi:10.1001/jamanetworkopen.2022.39131 36306129 PMC9617169

[21] Babroudi S, Weiner DE, Neyra JA, Drew DA. Acute kidney injury receiving dialysis and dialysis care after hospital discharge. J Am Soc Nephrol. 2024;35(7):962–971. doi:10.1681/asn.0000000000000383 38652567 PMC11230726

[22] Department of Health and Human Services. Applicability of ESRD PPS policies to AKI dialysis. Federal Register. 2016. Accessed April 15, 2025. https://www.govinfo.gov/content/pkg/FR-2016-11-04/pdf/2016-26152.pdf

[23] Vijayan A, Heung M, Awdishu L, ASN kidney health guidance on the outpatient management of patients with dialysis-requiring acute kidney injury. J Am Soc Nephrol. 2025;36(5):926–939. doi:10.1681/ASN.0000000646 40014384 PMC12059106

[24] Lou SS, Kim S, Harford D, Effect of clinician attention switching on workload and wrong-patient errors. Br J Anaesth. 2022;129(1):e22–e24. doi:10.1016/j.bja.2022.04.012 35568509 PMC9428910

[25] Johansen KL, Chertow GM, Gilbertson DT, US Renal Data System 2022 Annual Data Report: epidemiology of kidney disease in the United States. Am J Kidney Dis. 2023;81(3)(suppl 1):A8–A11. doi:10.1053/j.ajkd.2022.12.001 36822739 PMC10807034

[26] Centers for Medicare and Medicaid Services. ESRD quality incentive program. Accessed April 22, 2025. https://www.cms.gov/medicare/quality/end-stage-renal-disease-esrd-quality-incentive-program/measuring-quality

[27] Centers for Medicare and Medicaid Services. CMS ESRD measures manual for the 2025 performance period; November 14, 2024. Accessed April 22, 2025. https://www.cms.gov/files/document/esrd-measures-manual-v101.pdf

[28] Centers for Medicare and Medicaid Services. Dialysis facility compare. Accessed April 22, 2025. https://data.cms.gov/provider-data/topics/dialysis-facilities

[29] Struthers SA, Bieber SD. Outpatient hemodialysis for acute kidney injury post-Medicare coverage: how are we doing? Kidney Med. 2021;3(6):886–888. doi:10.1016/j.xkme.2021.08.007 34938996 PMC8664735

[30] Centers for Medicare and Medicaid Services. Innovation models: kidney care choices (KCC) model. Accessed April 22, 2025. https://www.cms.gov/priorities/innovation/innovation-models/kidney-care-choices-kcc-model

[31] Centers for Medicare and Medicaid Services. Kidney care choices (KCC) model. Accessed April 22, 2025. https://www.cms.gov/priorities/innovation/media/document/kcc-py23-rfa

[32] Barreto EF, Cerda J, Freshly B, Optimum care of AKI survivors not requiring dialysis after discharge: an AKINow Recovery Workgroup report. Kidney360. 2024;5(1):124–132. doi:10.34067/KID.0000000000000309 37986185 PMC10833609

[33] Neyra JA, Gewin L, Ng JH, Challenges in the care of patients with AKI receiving outpatient dialysis: AKINow Recovery Workgroup report. Kidney360. 2024;5(2):274–284. doi:10.34067/KID.0000000000000332 38055734 PMC10914193

[34] Organ Procurement and Transplantation Network (OPTN). Organ Procurement and Transplantation Network policies; 2024. Accessed March 23, 2025. https://optn.transplant.hrsa.gov/media/eavh5bf3/optn_policies.pdf

[35] McCoy IE, Silver SA. Clinical trials targeting recovery and post-discharge care in dialysis for acute kidney injury. Adv Kidney Dis Health. 2025;32(2):194–199. doi:10.1053/j.akdh.2025.01.008 40222806

[36] Hsu CY, Levy MC, Lawson ML. Beyond acute care for dialysis-requiring acute kidney injury: three patients’ stories and one nephrologist’s commentary. Adv Kidney Dis Health. 2025;32(2):111–114. doi:10.1053/j.akdh.2024.11.001 40222797

[37] Federal Register. Medicare program; end-stage renal disease prospective payment system, payment for renal dialysis services furnished to individuals with acute kidney injury, end-stage renal disease quality incentive program, and end- stage renal disease treatment choices model; 2022. Accessed February 16, 2025. https://www.federalregister.gov/documents/2022/11/07/2022-23778/medicare-program-end-stage-renal-disease-prospective-payment-system-payment-for-renal-dialysis 29091373

[38] Levy M Patient stories: Marla Levy. Accessed December 9, 2018. https://nephrology.ucsf.edu/patients/patient-stories/patient-stories-marla-levy

[39] Cook E; CBS News Bay Area. New UCSF study finds some diagnosed with kidney disease may not need dialysis [television broadcast]; December 21, 2023. https://www.cbsnews.com/sanfrancisco/news/ucsf-study-some-diagnosed-kidney-disease-may-not-need-dialysis/

[40] Leigh S Brief dialysis may be best for some kidney patients. UCSF News. September 28, 2023. Accessed January 15, 2025. https://www.ucsf.edu/news/2023/09/426271/brief-dialysis-may-be-best-some-kidney-patients

